# Spontaneous adverse drug reaction reporting by patients in Canada: a multi-method study—study protocol

**DOI:** 10.1186/s40064-016-1838-9

**Published:** 2016-02-29

**Authors:** Rania Al Dweik, Sanni Yaya, Dawn Stacey, Dafna Kohen

**Affiliations:** Population Health Program, Faculty of Health Science, University of Ottawa, Ottawa, ON Canada; School of International Development and Global Studies, Faculty of Social Science, University of Ottawa, Ottawa, ON Canada; Ottawa Hospital Research Institute, School of Nursing, Faculty of Health Sciences, University of Ottawa, Ottawa, ON Canada; School of Epidemiology, Public Health, and Preventive Medicine, University of Ottawa, Ottawa, ON Canada

**Keywords:** Adverse drug reactions (ADRS), Pharmacovigilance, Patient reporting, Canada

## Abstract

**Background:**

Monitoring adverse drug reactions (ADRs) through pharmacovigilance are vital to patient safety. Spontaneous ADR reporting is one method of pharmacovigilance, and in Canada all reporter types admitted to report an ADR to the Canadian Vigilance Program at Health Canada. Reports are submitted to Health Canada by post, telephone, or via the internet. The Canada Vigilance Program electronically records submitted information to detect medication safety alerts. Although previous studies have shown differences between patients and healthcare professionals (HCPs) on the types of drugs and reactions reported, relatively little is known about the importance of patient reports to pharmacovigilance activities. This article proposed a multi-method approach to evaluate the importance of patient ADR reporting on pharmacovigilance activities, by systematically review the available literature, comparing patient—versus HCPs-generated ADR reports that were submitted to the Canada Vigilance Program, and exploring patient views and experiences regarding the Canadian ADR reporting system.

**Methods:**

Guided by a risk-perception theoretical lens, the proposed multi-methods research study will involve three phases. Phase I is a systematic review of all studies that analyse the factors influence ADR reporting by patients to the pharmacovigilance schemes. Phase II is a descriptive statistical analysis of all ADR reports received by the Canada Vigilance Program database between 1 January 2000 and 31 December 2014 from patients and HCPs to compare ADRs reported by patients with those reported by HCP reports in terms of ADR seriousness, ADR classification by system organ class, and the medication involved based on the anatomical therapeutic class system. In phase III, an interpretative descriptive approach will be used to explore patient’s views and experiences on ADR reporting and usability of the Canadian Vigilance ADR report. Participants will be purposefully selected to ensure diverse backgrounds and experiences. Interviews will be digitally-recorded, transcribed verbatim, and inductively analysed to identify themes. Patients will be interviewed until theoretical saturation is achieved.

**Discussion:**

Findings from this research will highlight the role of the patients in directly reporting ADRs, and provide information that may guide streamline and optimizing patient ADR reporting. Policy makers, public health officials, and regulatory agencies will require this critical information in order to improve medication safety in Canada and worldwide.

## Background

Adverse drug reactions (ADRs) are a worldwide problem that affects all drugs and their users. They cause significant disability and mortality, and are expected to be associated with an economic drain on the healthcare system (Bates et al. 1995; Oshikoya and Awobusuyi 2009). ADRs are monitored in many countries and by the World Health Organization (WHO) since the 1960s using spontaneous reporting systems, also called ‘early warning’ systems (Stricker 2004). Pharmacovigilance is the science and activities relating to monitoring, detection, assessment, understanding, and prevention of ADRs (Aagaard et al. 2009). Monitoring product safety has been traditionally done by passive surveillance (voluntary reports) or the collection of spontaneously reported adverse events from healthcare providers and consumers following the administration of a medicinal product (Sharrar and Dieck 2013).

In the beginning of ADR monitoring, only doctors and dentists were allowed to submit ADR reports to these databases (Aagaard et al. 2009). But, because the health agencies started focusing more on patients’ safety, in 1995 all drug manufacturers, world widely, were mandated to report ADRs (WHO 2002). Later, other healthcare professionals (HCPs), pharmacists, and patients were allowed to report ADRs in hope that this would increase the volume and quality of ADR reports (Aagaard et al. 2009).

Spontaneous direct patient reporting may prove to be essential for continuous improvement and successful pharmacovigilance. However, the literature is deficient regarding the role of direct patient reporting, and its effects, on pharmacovigilance activities (Herxheimer et al. 2010). Much controversy remains among experts concerning the utility and efficacy of incorporating patient ADR reports into pharmacovigilance guidelines and protocols (Mitchell et al. 1988; Hazell et al. 2013a, b). Some researchers believe that patient ADR reporting is detrimental to pharmacovigilance activities while others fully support it and agreed that direct patient reporting is necessary to add that extra layer for good pharmacovigilance since patients as users of medications have first-hand knowledge of their experiences with ADRs (Aagaard et al. 2009; van Grootheest and de, Graaf L, de, 2003; Blenkinsopp et al. 2007).

To date, few countries, including Canada, recognize patient-submitted ADR reports in their pharmacovigilance activities (Aagaard and Hansen 2010). One of the studies showed that the quality of direct patient reporting has been found to be similar to that of reports from HCPs and the spontaneous patients reporting of serious adverse drug reactions were helpful in detecting drug alert (Aagaard et al. 2009; Blenkinsopp et al. 2007). Other study showed that compared with other sources, patients reported different categories of ADRs for different types of medicines (Aagaard et al. 2009). Patients, through their reports, can offer a more direct and precise view of what is actually occurring during the treatment period (Blenkinsopp et al. 2007). The reports from patients provide insight that reports from HCPs simply cannot. Unlike reports from physicians, these reports describe how the adverse effects affect people’s lives (Herxheimer et al. 2010). Patient ADRs have the potential to express descriptive details of the ADR.

Patients often differ in their risk perception with respect to drug therapy when compared to HCPs (Aronson 2006). In one of the studies that conducted in the UK, patients’ reports differ from HCPs reports in terms of categorising event types (Blenkinsopp et al. 2007). In another study participants were requested to provide their risk perception of ADRs associated with 13 different drug classes. The findings showed that the two groups perceived ADR risks differently. Among HCPs, anticoagulants and non-steroidal anti-inflammatory drugs [NSAIDs] were ranked as the most dangerous drugs with respect to ADRs, whereas patients did not rank NSAIDS among the most dangerous drugs and believed that aspirin [an NSAID] posed the least threat (Bongard et al. 2002). In Denmark, consumers were more likely to report ADRs from SOCs: ‘nervous system disorders’; ‘psychiatric disorders’ and ‘reproductive system and breast disorders’ (than other sources (Aagaard and Hansen 2010).

Furthermore, by allowing direct patients spontaneous reporting, we are allowing the patient to be actively involved in their treatment. With the added patient reports to those of HCPs, the spontaneous surveillance database can acquire data rich in additional information about ADRs (Hammond et al. 2007). It could be argued that this only speeds up the drug alert detection process.

The literature review has demonstrated a gap in knowledge of one of the earliest safety reporting system active since 1965. No studies were found that compare and contrast patient reports against HCP reports in Canada using adverse reaction reporting system of Canada. Understanding how patients are involved in ADR reporting can provide new insights into the importance and limitations of integrating patient-generated ADR reports into pharmacovigilance approaches which might help in improving medication use safety in Canada. This study aimed to provide new insights into the importance of patients ADR reporting. Policy makers, public health officials, and regulatory agencies require this critical information to improve medication safety.

### Objective and research questions

The objective of this protocol is to evaluate the importance of patient ADR reporting on pharmacovigilance activities, by systematically review the available literature, comparing patient—versus HCPs-generated ADR reports-based on seriousness, system organ class—anatomical therapeutic class—that were submitted to the Canada Vigilance Program, and exploring patient views and experiences regarding the Canadian ADR reporting system. The study will involve three phases that will focus on the following *research question*s:

1. What are the factors influencing reporting of adverse drug reaction by patients?
2. What types of ADRs, suspect drug classes, reaction severity, and ADR-involved organ systems are reported by patients versus HCPs?
3. What are the views and experiences of patients regarding ADR reporting and the user-friendliness of ADR reporting in Canada?

### Conceptual framework

The *Risk Perception Theory* will provide a theoretical lens to examine the differences between patients and HCPs perceptions in term of the number of serious ADRs reported by each group. It works on the concept that different classes of people [professionals versus non-professionals, scientists versus the public, or, in this case, HCPs versus patients] have different views on the possible risks associated with some action or environment (Af WÅhlberg AE. 2001).

## Methods

This study protocol consists of three complementary phases, each phase sequentially informs the next.

### Phase I: Systematic review of all studies that analyse the factors influencing reporting ADRs by patients to the pharmacovigilance systems

*Research question 1* What are the factors influencing reporting of adverse drug reaction by patients?

### Methods

A systematic literature review will be guided by the Cochrane Handbook (Higgins and Green 2011), and will be structured to meet the Preferred Reporting Items for Systematic Reviews and Meta-Analyses (PRISMA) (Moher et al. 2009) criteria. Eligible studies addressed the factors influencing ADR reporting by patients. The search strategy protocol will be developed collaboratively with an academic reference librarian to refine queries and characterize them in terms of elements. Study inclusion/exclusion criteria will be framed in a systematic manner, using the elements of a question and include population, intervention, comparator, and outcome (PICO) (Stillwell et al. 2010) (Table 1) even if all these elements are not used in the formal search strategy.

**Table 1:** Systematic review inclusion/exclusion criteria

	Include	Exclude
Study Design	Regardless of methodology, eligible studies answered the question: what factors influence reporting ADRs by patients?	–
Population	Studies addressing patients view and perceptions on ADRs reporting	Studies addressing: the view and perception of HCPs on ADRs reporting, the role of HCPs in ADR reporting, ADRs reported by HCPs, restricted to specific drugs or therapeutic class
Setting	No restriction on setting	–
Language	All studies in English. Non-English studies will be converted to English using Google Translate (due to resources limitation)	–

#### Search strategy

The search will focus on all major databases relevant to the subject matter, i.e., PubMed, MEDLINE, EMBASE, CINAHL, PSYCINFO, ProQuest Dissertation and Theses, and Cochrane library. These databases will be searched using both medical subject heading (MeSH) and text search terms including: patients, consumers, public, adverse drug reactions, report, reporting, spontaneous, pharmacovigilance, and surveillance. The search dates will include all years for the database, i.e., from the earliest data sources on each database, e.g., 1947 or earlier, and up to December 2014. The reference lists of each included study will be checked to identify additional studies for potential inclusion. Grey literature searches will be performed using similar search terms.

#### Data extraction

All data from the included studies will be extracted using a data extraction form. The form will be pilot tested prior to commencing data extraction. After the identification of studies, duplicates will be removed and two independent reviewers will conduct three levels of screening. Level one of screening is to determine study relevance to the overall objective of the systematic review and this will include title screening. Level two screening will be a title and abstract screen based on the inclusion/exclusion criteria. Level three screening will include reviewing the final group of studies identified by both reviewers as meeting the inclusion criteria. A second reviewer will verify the accuracy of extracted data. Study data quality and study bias risk assessments will be performed. The two independent reviewers will assess the quality of included studies using the appropriate critical appraisal skills programme (CASP) checklist (Milne and Oliver 1996).

#### Data analysis

The data will be synthesized by classifying the different study types, and by comparing and contrasting study findings. Characteristics of included studies will be analysed descriptively and the results will present in a narrative format recommended by the PRISMA (Preferred Reporting Items for Systematic Reviews and Meta-Analyses) statement (Liberati et al. 2009).

#### Bias

A prior plan was made to minimize the risk of bias (Hartling et al. 2009) using Assessment of Multiple Systematic Reviews (AMSTAR) (Shea et al. 2009). The systematic review protocol was established, and the two researchers will be involved in screening the identified literature and will participate in data extraction.

### Phase II: Observational retrospective descriptive statistical comparison of ADR reports from patients versus healthcare professionals

#### Research questions and hypothesis

1. Are there a significant difference in distribution of ADRs between reports (patients vs. HCPs) based on ADRs seriousness)? Does the trend change?
2. Are there significant differences in distribution of ADRs between reporters (patients’ vs HCPs) for system organ classification (SOC)?
3. Are there significant differences in distribution of ADRs between reporters (patients’ vs HCPs) for the suspected drug class involved?

### Methods

#### Study design

An observational—retrospective cross-sectional study design will be used. The study will include all ADR reports submitted to the Canadian Vigilance Database from January 2000 through December 2014, inclusive. Using 14 years data will help to observe the changes in patients reporting. The ADR report analysis will compare content from patients—versus HCPs-submitted reports in terms of ADR seriousness, ADR classification by system organ class, and the medication involved based on the anatomical therapeutic class system (ATC). Only ADRs classified as ‘serious’ will be analysed because identifying serious ADRs are the primary focus of spontaneous reporting systems and are of particular public health interest. The unit of analysis will be the ADR.

#### Study setting

The Canada Vigilance database contains all spontaneous ADR reports in Canada that were reported directly by HCPs, patients, and pharmaceutical companies. An ADR report is defined by the following four criteria, which must be included in all reports: (1) patient information; (2) the suspected medicine(s); (3) patient outcome information (s); and (4) reporter. Reports are categorized in the database by the degree of seriousness according to the Council for International Organizations of Medical Sciences criteria (Health Canada 2007). Health Canada Vigilance handles the ADR reports from the patients the same way it handles reports from other sources. The Canadian Vigilance database defines the following four categories of persons submitting reports to the database as: (1) *community* including patients, patients’ relatives, other members of the public; (2) *hospital* including HCPs such as physicians, pharmacists, and nurses; (3) *MAHs* including pharmaceutical companies; and (4) *others* including insurance companies.

#### Data extraction

Anonymized data will be extracted from the Canadian Vigilance ADR database based on the date reports were received, category of person submitting the reports, reaction seriousness, medications involved, and category of ADRs classified by SOC. The reported ADRs were coded according to the Medical Dictionary for Regulatory Activities (MedDRA) structure on SOC. The Canadian Vigilance database ADR search criteria include either the trade names or generic name of medications reported to cause ADRs. Therefore, it will be necessary to manually translate the trade names and generic names in the pharmacological/therapeutic subgroup of ATC level 1 to present the ADR data in a comprehensive format (Aagaard et al. 2009).

#### Medical dictionary for regulatory activities (MedDRA)

The Medical Dictionary for Regulatory Activities (MedDRA) was developed by the International Conference on Harmonization (Journot et al. 2008). It is clinically validated international medical terminology used by regulatory authorities and the regulated biopharmaceutical and pharmaceutical industries (MedDRA 2009). MedDRA is needed for electronic exchange of information on adverse reactions between industry and regulatory authorities after drug registration in the European Union (Brown 2004). The terms in MedDRA are numerically coded to support electronic means of data transmittal and retrieval. It has 26 broad groups called system organ classes. MedDRA was used for coding ADR reported by HCPs and patients in this study. The SOC will be identified via this method and compared according to reporter.

#### Anatomical therapeutic chemical system (ATC)

The Anatomical Therapeutic Chemical (ATC) system classifies medicinal products according to their primary constituent, the organ or system on which they act, and their chemical, pharmacological and therapeutic properties (Aagaard et al. 2009). Medicinal products are classified into groups on five different levels. The medicines are divided into 14 main groups (first level), with one pharmacological/therapeutic subgroup (second level). The third and fourth levels are chemical/pharmacological/therapeutic subgroups, and the fifth level is the chemical substance. The second, third, and fourth levels are often used to identify pharmacological subgroups if this is considered more appropriate than the therapeutic or chemical subgroups (Methodology WCCfDS 2011). In this study, ATC level one will be used to comprehensively categorize ADR data into one of 14 groups.

#### Data analysis

The main goal of the statistical analysis is to investigate the dependence between the reporter and the reported ADR (classified by seriousness, SOC, and ATC) using the “null hypothesis”. It will be assumed that there are no differences in the number of serious ADRs reported by patients versus HCPs. In case the null hypothesis is rejected and to ensure that any reporting differences between patients and HCPs will be indeed significant and not due to chance, the data will be analysed using the Chi-squared test for independence. This test is used to determine whether the frequency of each group is different from what would be expected. If the calculated Chi-square is high enough, then the frequencies found would not be expected on the basis of chance and the null hypothesis will be rejected. Odds ratios (ORs) will be calculated using a 2 × 2 table for the reporter category (patients and HCPs), SOC, or ATC. Confidence intervals will be calculated for all ORs (95 % level). Statistical analyses will be performed using SPSS version 17 statistical software.

### PHASE III: Explore patient views and experiences on reporting ADRs to the Canadian vigilance database

*Research question 3* What are the factors influencing patient ADR reporting? What are the views and experiences of patients regarding the user-friendliness of ADR reporting in Canada?

### Method

#### Study design

An interpretive descriptive qualitative study will be conducted. Interpretive description is an inductive and effective analytic approach for describing health care events (Thorne et al. 2009).

#### Participants and setting

Eligible participants will be adult patients over 18 years old living in Canada, have experienced an ADR, and able to answer questions in English or French. Purposeful sampling will be used to represent a range of ages (18–45, 45–64, 65–80), sex (male, female), and patients who have reported a side effect to pharmacists, physicians, nurses, or to the Canada Vigilance Database. If we encounter difficulty-finding participants, we will revert to the snowball technique. This approach is a sampling strategy that consists of seeking referrals from participants, where one participant gives the researcher the name of more possible participants (Creswell 1998). This technique is used in populations where it is difficult to identify potential participants. Recruitment will continue until data saturation plus an additional three participants, to ensure that no new themes will be identified (Francis et al. 2010). We anticipate reaching saturation with 10 participants and therefore recruiting a total of 13 patients.

*Participant recruitment* Patients will be informed about study details and invited to participate through social media (e.g. Kijiji, Facebook, twitter), patient associations, and patient societies such as Patients Canada, Canadian Arthritis Society, or Canadian Cancer Society or Heart & Stroke.

#### Data collection

First, an email or pamphlet introducing the study will be given to prospective participants that express an interest in the study. Those willing to participate will have an interview set up at a convenient time and location (including in person, telephone, or skype). During the interview, the researcher will explain the purpose of the study, describe the approximate amount of time it will take to complete the interview, and inform the participant that they can withdraw from the interview at any time or refuse to answer any questions. Participants will also be informed of precisely how their data will be used, and an informed consent form will be provided for approval, signature, and return. The interview will be conducted using a semi-structured interview guide and is expected to take 30–45 min. The semi-structured interview guide was developed based on key sensitizing concepts from the literature on ADR reporting and with review by experts in pharmacy, knowledge translation to patients, and health policy. More specifically, the first set of questions will explore participant experiences with medication-related side effects, severity, who they report side effects to, and expectations about reporting. The second set of questions will focus on the user-friendliness of the ADR reporting form that is available on the Canada Vigilance website. The interviewer will lead the patient through the form step-by-step and then ask questions exploring the clarity of the questions, ease of use, readability, and font size. Non-identifying demographic information will also be collected. Interviews will be audio-taped and field notes documented by the researcher.

#### Data analysis

Interview audiotapes will be transcribed verbatim and analysed. Participant responses to the semi-structured interview questions will be analysed using interpretive content analysis (Huberman and Miles 1994). Interpretive content analysis is an iterative process that facilitates analysis of text that describes processes, activities, perceptions, and beliefs (Thorne et al. 2009). The qualitative data analysis process will use the three-step method of data reduction, data display, and drawing conclusions/verification (Huberman and Miles 1994). Several techniques will be used to enhance the credibility, auditability, and transferability of the study findings (Lincoln and Guba 1986).

To enhance credibility, several techniques will be used including: (a) use of open-ended interviewing techniques: tape recording and verbatim transcriptions to increase the accuracy of describing each participant’s experience;(b) share interpretation of findings with participants to enhance accuracy from the perspective of the person’s living experience; and (c) extensive literature review (Lincoln and Guba 1986).

Auditability is the second assessment of accuracy (Lincoln and Guba 1986). This will be enhanced through presentation of enough information to the reader to see how the raw data lead to the interpretation.

The third criterion of assessment is transferability of the data by presenting enough details on the finding that will allow others in the discipline to determine the relevance of the study finding for their own practice, research, and theory development.

## Strengths and limitations

Using secondary data from the official website of the Health Canada database in Phase II to compare serious ADRs reported by HCPs versus patients is considered cost-effective and time-efficient. Since the database contains a large number of ADR reports; sample size is not a problem. But because there is no limitation on the ATCs in the database, this would result in a broader scope of information that will be obtained and more manual work will be needed. One of the study limitation that needs to be addressed is the fact that one reporter can report more than one ADR per report, thus resulting in ADRs not being truly independent. It is important to note that when analysing data obtained from the Canada Vigilance Database, causality of a reaction by any given suspect drug cannot be assumed. In addition, comparisons between drugs cannot be made from the available data (Health Canada 2011).

Using the interpretive descriptive approach in Phase III will create an opportunity to understand the patient perception toward adverse drug reaction reporting and the Canadian Vigilance system which will help in interpreting and understanding the results from phase II. (Thorne et al. 1997). In phase III, efforts to include participants with various demographic characteristics, an attempt to broaden the recruitment, will be conducted by providing posters to patient associations, and use advertisements and Internet resources to facilitate participation.

### Ethic

Secondary data obtained from the official website of Health Canada will be used in this research. Secondary data analysis does not pose a privacy risk to participants the way that primary data collection and analysis might, but the rights of participants will still need to be protected. All patient data will be anonymized and this study protocol had received approval of the University of Ottawa Research Ethics Boards in July 2014 (H05-14-18).

The ethical aspects of the research involving human participants will follow the guidelines of the national granting councils (the Canadian Institutes of Health Research) set out in the Tri-Council Policy Statement on Ethical Conduct for Research Involving Humans (Ethics PoR. Tri-Council Policy Statement: Ethical Conduct for Research Involving Humans (TCPS) 2001).

For ethics approval, four major ethical concerns were considered: risk, benefit, consent, and confidentiality. The study is not anticipated to involve direct risk to participants because no active intervention will occur. Participants will be aware that participation will be voluntary and that they are free to skip any question(s) that make them uncomfortable, and can withdraw from the study at any time.

Benefit will be explained as an opportunity to inform the scientific community and policy makers on current ADR issues and concerns. After participation, interviewees will be given the opportunity to ask about current reporting procedures to gain knowledge on the subject. There is no financial benefit to study participants. Each participant will provide written informed consent prior to participate in the study. The consent form will be explained prior to beginning the study and questions will be answered. Participants will be informed that steps to ensure confidentiality will be taken. All interviews will be conducted in a one-on-one manner to augment participant confidentiality. Participants will be identified in all written reports by numbers only, not by names. Any identifying information will be removed from the transcripts and all electronic files will be kept password protected, while hardcopy files will be kept in a locked cabinet at researcher student office—Health Science Department—University of Ottawa.

## Discussion

Adverse drug reaction reporting systems need to be robust in order to be able to detect new drug alerts and improve pharmacovigilance. These systems are necessary to identify new reactions and to prevent potential ADRs. The purpose of this research is to enhance the understanding of safety issues associated with medication use.

The methodology proposed here has several strengths. First, we will systematically review all published studies that involve patients reporting to national agencies and compare that with what has been done in Canada. Second, we will descriptively analysis all ADR reports received by the Canada Vigilance Program database between January 1st 2003 and December 31st 2012 from patients and HCPs and compare these submitter groups in terms of: seriousness of suspected ADRs reported, distribution of ADR reports based on affected organ system(s), and the suspected medicine classes involved. And third, we will use an interpretative descriptive approach to explore patients’ views and experiences on ADR reporting and usability of the Canadian Vigilance ADR report. This will create an opportunity to understand patient perceptions toward the Canadian Vigilance system and will help in interpreting and understanding the results from descriptive analysis of the data collected. One of the advantages of using the interpretive descriptive methods is the ability to collect accurate data and provide a clear picture of the description under study (Ethics PoR 2001).

Findings from this research will make three significant contributions: (1) it will highlight the role and process of patients in directly reporting ADRs; (2) give new information that may guide streamline in optimizing patient ADR reporting and; (3) provide policy makers, public health officials, and regulatory agencies with this critical information in order to improve medication safety in Canada.

## Abbreviations

ADR: adverse drug reactions
HCPs: healthcare professionals
ATC: anatomical therapeutic chemical
SOC: system organ classification
CASP: critical appraisal skills programme (Milne and Oliver 1996)
AMSTAR: assessment of multiple systematic reviews
